# Visually Mediated Odor Tracking During Flight in Drosophila

**DOI:** 10.3791/1110

**Published:** 2009-01-26

**Authors:** Mark A. Frye, Brian J. Duistermars

**Affiliations:** Department of Physiological Science, University of California, Los Angeles

## Abstract

Flying insects use visual cues to stabilize their heading in a wind stream. Many animals additionally track odors carried in the wind. As such, visual stabilization of upwind tracking directly aids in odor tracking. But do olfactory signals directly influence visual tracking behavior independently from wind cues? Additionally, recent advances in olfactory molecular genetics and neurophysiology have motivated novel quantitative behavioral analyses to assess the behavioral influence of (e.g.) genetically inactivating specific olfactory activation circuits. We modified a magnetic tether system originally devised for vision experiments by equipping the arena with narrow laminar flow odor plumes. Here we focus on experiments that can be performed after a fly is tethered and is able to navigate in the magnetic arena. We show how to acquire video images optimized for measuring body angle, how to judge stable odor tracking, and we illustrate two experiments to examine the influence of visual cues on odor tracking.

**Figure:**
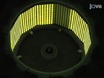


## Protocol

The OMT is an adaptation of a magnetic tether system ^1^ designed to incorporate a virtual plume simulator ^2^. The following protocols will explain how to optimize the acquired video images **(Part 1)** and run two types of basic visuo-olfactory experiments **(Part 2 & 3)**.

### Part 1: Video Acquisition

Optimizing the video image is crucial for the quality of the acquired data. Follow these steps for cleaner images.

Place a freshly tethered fly in the arena using a pair of forceps.
Position the firewire board camera (Fire-I 1394store.com) under the clear acrylic vacuum chamber such that the camera view is directly upward through the chamber, through a glass tube, to the ventral aspect of the tethered fly. Be sure that there is no IR filter coating on the lens of the video system.
To illuminate the fly, use a circular array of infrared LEDs (Small-Parts Inc) positioned just under the LED panels. Focus each LED on the fly’s body individually for uniform illumination.
To reduce glare from the panel system and to minimize visual cues observed by the fly, it is useful to coat all reflective surfaces within the camera view with flat black paint. Room lights should be switched off to reduce glare and brightness modulations detectable by the fly’s eye.
Visually inspect the camera image. The fly should be bright white against a black background. If the image is blurred the lens may need to be focused. If the fly is dark, the LED array may be unfocused or insufficiently powered for proper illumination. If the background contains bright objects, mount a disk of black flock paper just above the fly to minimize background reflectance.
We acquire video with custom subroutines written in Matlab using the Image Acquisition toolbox.

### Part 2: The fly finding the odor plume

One simple experiment in the OMT is observing a hungry (starved) fly locating and then actively tracking a plume of attractive food odor. Manipulating the visual surroundings influences the fly’s ability to robustly track an appetitive food odor. Note that experiments are typically run in a random block format to minimize any bias in experimental order. All external hardware (gas multiplexers, LED arena, video acquisition) are controlled by custom software routines written in Matlab (Mathworks).

Place a freshly tethered fly in the arena using a pair of forceps.
Before every experiment, using the visual display, rotate the fly for several revolutions ^3^ to ensure smooth motion around the entire 360 yaw axis. If the fly does not spin smoothly discard it. If the problem persists, the likely cause is misaligned magnets. Note: If the distribution of arena heading for several trajectories during the spin trial shows significant peaks (i.e. is not flat over 360 degrees), there is likely an orientation bias due to misaligned magnets.
Present the fly with the following sample experimental conditions in random order: i) high-contrast striped pattern with water vapor emanating from the odor port; ii) high-contrast striped pattern with attractive odor vapor; iii) a single vertical stripe offset 90 degrees from the water vapor nozzle; and iv) a single 90 degree offset stripe with odor vapor. In our experience, 30 second intervals work well for experiments where flies are challenged to locate the odor source on their own. We acquire video at 30 Hz for the duration of each experimental condition and analyze the images offline using custom software routines written in Matlab (one of the most useful built-in Matlab functions is regionprops.m which analyzes the geometric properties of an ellipse.
When using high odor concentrations, residual odor may be released from the odor port after the odor is switched off. To minimize any effects this may have it is useful to spin the fly for a few seconds with a rotating panorama to allow the system to flush out any residual odor.
It is also beneficial to visually rotate the fly between experimental treatments to (i) keep the flies actively engaged in the experiment and to (ii) keep flies from remaining in one spot. An example experiment may have the following order: i) twenty second initial diagnostic spin; ii) five second intermediate spin; iii) twenty second condition pair #1; iv) five second intermediate spin; v) twenty second condition pair #2.

### Part 3: The fly remaining within the odor plume

A second basic experiment in the OMT is to visually drag a fly into an odor plume, instantaneously change the visual conditions, and then measure its ability to remain in the plume. Again, note that all experiments are done in a random block format to minimize any bias in experimental order.

Place a freshly tethered fly in the arena using a pair of forceps.
Visually rotate (spin) the fly several times around the arena before every experiment as a diagnostic check to ensure that the animal has the capacity to orient in every direction (see **Part 2.2**).
Before each condition, turn on the experimental odor (water or vinegar) and visually “drag” the fly into the plume by oscillating a small vertical stripe at the position of the plume. This takes advantage of the powerful stripe fixation behavior observed in flies [4].
Now that the fly is oriented toward the plume, remove the oscillating stripe and present the following sample visual conditions: i) high contrast panoramic stripes with water vapor (**Fig. 1A**); ii) uniform background with water vapor (**Fig. 1B**); iii) high contrast panoramic stripes with odor vapor (**Fig. 1C**); iv) uniform background with odor vapor (**Fig. 1D**). Acquire video as in **2.3**. An example experiment may have the flowing order: i) twenty second initial diagnostic spin; ii) five second plume stripe oscillation; iii) twenty second condition pair #1; iv) five second plume stripe oscillation; v) twenty second condition pair #2.

### Representative Results:


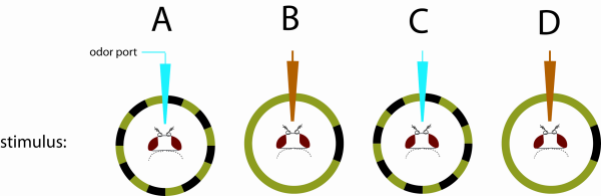


**Figure 1**

Part 1 explains how to optimize the OMT video images. The video image should have a clear view of the illuminated fly on a dark black background. Overly bright images can be improved by reducing background interference, reducing the intensity of the IR LEDs, or by turning off the room lights. If the fly is improperly illuminated, adjusting the focus of the IR LEDs or modulating the intensity of the IR LEDs may fix the problem. **Parts 2 & 3** describe simple experiments to conduct to verify the proper operation of an OMT. In all cases, a fly should be visually “forced” (via optomotor responses) to rotate several revolutions in the arena to ensure a smooth and complete range of motion. In **Part 2** a fly is challenged to find an odor plume on its own. To keep the fly engaged in the experiment, to reduce the effect of residual odor release, and to keep flies from maintaining a single heading throughout the experiment, it is useful to visually rotate the fly for a few seconds between trials. A fly should never localize a water vapor plume to a significant degree, and should always localize an odor plume in the presence of rich panoramic visual cues. In **Part 3** a fly is visually dragged directly into an odor plume and challenged to maintain its heading within the plume against a variety of stationary visual backgrounds. If the fly is dragged into a water vapor plume, it should quickly turn away. If the fly is dragged into an odor plume and there are sufficient visual cues to mediate robust tracking, the fly will remain in the plume.

## Discussion

The image acquisition for this system utilizes an inexpensive firewire board camera and software written in Matlab. Clear images are crucial for obtaining accurate data traces. The strategies described above are useful for cleaning up images in this system. Other video tracking procedures for this system have been described ^1^. Furthermore, this system could easily be modified to include real-time video tracking. The example experimental protocols described here meant to be a starting point to ensure the proper operation of an OMT. It is possible to experiment with different visual displays and even different odors.
